# Humans can identify reward-related call types of chickens

**DOI:** 10.1098/rsos.231284

**Published:** 2024-01-03

**Authors:** Nicky McGrath, Clive J. C. Phillips, Oliver H. P. Burman, Cathy M. Dwyer, Joerg Henning

**Affiliations:** ^1^ School of Veterinary Sciences, University of Queensland, Gatton, Queensland 4343, Australia; ^2^ Institute of Veterinary Medicine and Animal Science, Estonia University of Life Sciences, Tartu, Estonia; ^3^ Curtin University Sustainable Policy (CUSP) Institute, Kent Street, Bentley, Western Australia 6102, Australia; ^4^ School of Life Sciences, University of Lincoln, Brayford Pool, Lincoln, Lincolnshire LN6 7TS, UK; ^5^ Scotland's Rural College (SRUC), Peter Wilson Building, Kings Buildings, West Mains Road, Edinburgh EH9 3JG, UK

**Keywords:** vocalization, reward, chicken, human

## Abstract

Humans can decode emotional information from vocalizations of animals. However, little is known if these interpretations relate to the ability of humans to identify if calls were made in a rewarded or non-rewarded context. We tested whether humans could identify calls made by chickens (*Gallus gallus*) in these contexts, and whether demographic factors or experience with chickens affected their correct identification context and the ratings of perceived positive and negative emotions (valence) and excitement (arousal) of chickens. Participants (*n* = 194) listened to eight calls when chickens were anticipating a reward, and eight calls in non-rewarded contexts, and indicated whether the vocalizing chicken was experiencing pleasure/displeasure, and high/low excitement, using visual analogue scales. Sixty-nine per cent of participants correctly assigned reward and non-reward calls to their respective categories. Participants performed better at categorizing reward-related calls, with 71% of reward calls classified correctly, compared with 67% of non-reward calls. Older people were less accurate in context identification. Older people's ratings of the excitement or arousal levels of reward-related calls were higher than younger people's ratings, while older people rated non-reward calls as representing higher positive emotions or pleasure (higher valence) compared to ratings made by younger people. Our study strengthens evidence that humans perceive emotions across different taxa, and that specific acoustic cues may embody a homologous signalling system among vertebrates. Importantly, humans could identify reward-related calls, and this ability could enhance the management of farmed chickens to improve their welfare.

## 1. Introduction

Sounds are produced in mammals, reptiles and amphibians, when airflow from the lungs passes into the larynx—the airflow makes the vocal folds vibrate, causing sound waves to leave the mouth. These vibrations of the folds determine the fundamental frequency [1] and the first harmonic of the sound [2–5]. In birds, a similar process occurs; the syrinx, the vocal organ at the base of the trachea, is the source of the vibration generating the sound, which is then modified by the suprasyringeal vocal tract [6,7].

The ability to decode emotional information contained within the vocalizations of other species provides an adaptive benefit to animals [8]. Being able to decipher the emotional state in an alarm call helps receivers to determine the severity of the threat and is particularly advantageous in dangerous situations. Similarly, situations where receivers or senders would benefit from the accurate perception of emotional states within species include territory disputes, avoidance of predators, social interactions and the survival of newborns [9,10].

Emotional excitement can result in laryngeal muscle tension which affects air flow through the vocal tract and thereby impacts the acoustic parameters of vocal sounds [11,12]. The emotions of non-human animals are often decoded by reference to arousal (from excitement to content or low to high arousal) and valence (from negative emotions/displeasure to positive emotions/pleasure) [13,14].

Increases in arousal generally produce vocalizations that are harsher, louder, faster and longer, with a higher frequency (*f0*) and a wider frequency range [15].

Vocal correlates of valence are less easy to define, though positive contexts mainly elicit shorter call durations [16–18]. A growing body of studies has revealed that these acoustic properties appear to accurately predict human ratings of arousal and, to a lesser degree, valence in some animals, including dogs [19–23], pigs [24], silver foxes [25] and wild and domestic ungulates [26].

In two studies, the success of participants in correctly classifying and describing emotionality in pig *Sus scrofa* and dog *Canis familiaris* calls, respectively, was attributed to use of Morton's motivation-structural rules [20,24]. These rules dictate that a call's frequency [27] is determined by the context it is produced in: high frequency calls are produced in fearful or appeasing contexts, whereas low frequency calls represent aggressive contexts. This has been validated in various species, including dogs [28], chimpanzees *Pan troglodytes* [29], coatis *Nasua narica* [30] and elks *Cervus elaphus* [31].

These studies suggest that there are cross-taxa similarities in how emotions are conveyed acoustically and perceived across mammalian groups [27,32–35], and these allow humans to correctly assign emotional contexts to calls produced by other species. There is some evidence for this in more distantly related species, including birds, reptiles and amphibians [36], e.g. hour-glass treefrogs *Dendropsophus ebraccatus*, American alligators *Alligator mississippiensis*, black-capped chickadees *Poecile atricapillus* and common ravens *Corvus corax*. However, this has so far only been shown with negatively valenced emotions, such as those connected to distress, fear aggression and defeat [37]. To date, no studies have considered whether humans are able to correctly identify animals' reward-related vocalizations, which would represent positively valenced emotions.

It is also not known if the accuracy of human interpretation of animal calls is influenced by familiarity with the species. Some studies found that call context recognition was enhanced by experience with the target species [38,39], while others have found that experience did not aid recognition of call contexts [21,22,40]. Scheumann *et al*. [41] concluded that humans rely more on experience-dependent cognitive mechanisms than induced emotional state or acoustic cues to recognize the emotional content of vocalizations.

This study investigated whether humans can correctly identify calls made in reward or non-reward context by another vertebrate animal, the domestic chicken, which is the most commonly farmed species in the world. Domestic chickens also have a wide and varied vocal repertoire with up to 25 discrete calls documented in various studies [42–44]. Correct identification could be useful for chicken farmers to determine the welfare of their animals. We hypothesized that the ability of humans to correctly identify the context of calls, especially positive contexts, would be modified by experience with chickens, with those having more experience with chickens making more correct identifications.

## 2. Methods

### 2.1. Experimental study to record chicken vocalizations

#### 2.1.1. Acoustic vocalizations of chickens

Vocalizations were generated using calls recorded during a previous experiment by McGrath *et al*. [18]. These vocalizations were elicited by sound cues signalling rewards, or during two non-reward (control) treatments (sound cue—no reward and no sound—no reward). Twelve ISA brown hens, approximately 18 weeks old, were subjected to a Pavlovian conditioning paradigm. An initially neutral stimulus (conditioned stimuli, CS) was repeatedly paired with the presentation of one of three different rewards (mealworms, normal food or a dustbathing substrate), or a sound-neutral event (an empty compartment) which served as an unconditioned stimulus. Hens were placed individually in the first chamber of an experimental pen with two chambers. Vocalizations were recorded during a 15 s period after a sound cue was played to signal the availability of a dustbathing substrate, mealworms or normal food in a second chamber. After the recording period, a light was switched on to signal the door had been unlocked, and hens were able to push through the swing door to access the rewards. Non-reward vocalizations were recorded during the sound-neutral event (CS paired with an empty compartment) and a ‘muted-neutral’ treatment (no sound cue, empty compartment).

#### 2.1.2. Sound recordings

All vocalizations were recorded using a microphone (Sennheiser ME66 condenser shotgun) and a data recorder (Tascam DR100 MkII DAT). Gain settings were set to high while the rotary dial input gain setting was set at 6. Recordings were conducted with 24 bit resolution at a sampling rate of 44.1 kHz. The DAT files were transferred to a PC (Dell) to analyse the vocalizations using Raven Pro: Interactive Sound Analysis Software (Version 1.5, Cornell Laboratory of Ornithology, Ithaca, NY, USA).

During recording, hens produced a variety of different call types, and four were used as stimuli for the cross-sectional study. Two were produced in anticipation of rewards, the ‘food’ call and the ‘fast cluck’, and two other call types were produced in non-reward contexts, the ‘whine’ and the ‘gakel’ call. In total, 16 recordings were selected, one of each call type from four hens. Eight of these calls had been made in the rewarded context and eight were made in the non-rewarded context. Selection of recordings for use in the cross-sectional study was based on the quality of the recordings available, minimizing background noise as much as possible. All playback calls were edited using Adobe Audition CC sound editing software [45].

Call duration is a measure of time (s) from the beginning of the first syllable in a sequence to the end of the last syllable in a sequence and has been demonstrated to vary according to differences in arousal or valence in some animals [15]. For each call used in the survey, a box around each syllable (defined as a continuous impression in time on the spectrogram recordings) was created in Raven Pro 1.5 to measure the duration of the call.

The average (standard deviation) call lengths for each of the call types were as follows: food call 0.89 s (0.10 s); fast cluck 0.88 s (0.08 s); whine 1.76 s (0.20 s); gakel call 3.77 s (0.44 s).

However, in order to create a standardized 6 s playback length for each call to participants, the shorter calls (food calls and fast clucks) were looped.

Samples were down-sampled to 22.5 KHz, normalized to −26 dB root mean square, and saved as wav files, before being converted to mp3 files for use in the survey. We used mp3 files as this file format is compatible with most of the Internet browsers in which the online questionnaire was completed.

### 2.2. Cross-sectional study on human recognition of chicken calls

#### 2.2.1. Participant selection

Participants in this study were recruited using a ‘virtual snowballing’ technique which involved requesting personal and professional colleagues of the research team (by email, or through Twitter or Facebook) to complete an online questionnaire. The initial message asked contacts to forward a link to an online questionnaire to their personal and professional contacts. A link to the online questionnaire was also posted in an article in an online industry journal (eChook, PoultryHub). Sample size was calculated to estimate the proportion of participants that correctly identify the context in which a call was made. As the expected sample proportion was unknown, it was set to 50% to maximize the sample size. Using a 95% confidence interval, a precision of 7% and an estimated population of 1000 of people viewing the survey, a sample size of 165 participants was predicted to be sufficient.

#### 2.2.2. Questionnaire design

The online questionnaire was accessed through online software [46]. Participants were informed that they must be 18 years or older, that participation was voluntary, and that all responses would remain anonymous.

Before listening to any sounds, participants were informed that the study investigated how people perceive information contained within animal vocalizations. They were then told that they would hear a number of calls made by chickens.

Following this was a short explanation about the scales that the respondents would need to use to rate the calls. The two scales used were an ‘emotional scale’, which represented the valence of the emotion (from high displeasure (negative) to high pleasure (positive)), and an ‘arousal scale’, which represented the level of arousal the participants thought the hens were experiencing (from low to high arousal). The mid points of both scales represented a neutral rating. Participants were asked to take 10–15 min to complete the survey, and informed that, by continuing to the next page, they were consenting to take part in the survey, that they could withdraw at any time, that their response was anonymous and would be treated as confidential. A link provided further information about the survey, ethics clearance and contact details.

Participants then listened to 16 individual sounds presented in a random order using the ‘Randomize Pages' tool on SurveyGizmo. They were asked to rate each sound according to the level of emotional properties (valence) and level of excitement (arousal) they thought the chickens were experiencing, using the visual analogue scale from low to high. Participants were also asked whether the calls were made in a rewarded context or not (reward/no reward).

The final section requested demographic information, including age (<25, 25–34, 35–44, 45–54, 55–64, 65+years), gender (male, female), continent of origin (Europe, North America, South America, Australasia, Africa, Asia), final education level (primary, secondary, certificate, diploma, undergraduate, postgraduate) and whether they currently lived in an urban, suburban or rural environment. Participants were also asked whether they had had experience with chickens in the following five categories: (i) working in the industry, (ii) scientific research with live chickens, (iii) keeping chickens at home, (iv) interacting with chickens outside home or the workplace, or (v) any other experience with chickens, their length of the experience in each category (none, up to 1 year, 2–5 years, 6–10 years, more than 10 years), and their current rate of contact with chickens (none, once or twice a year, once or twice a month, once or twice a week, or several times a week). Finally, they were asked whether they currently owned a pet, and whether they had owned a pet during their childhood.

#### 2.2.3. Study population

A total of 351 participants accessed the online survey. Those who did not complete the survey were excluded and 194 complete responses were analysed. Each respondent evaluated all 16 chicken calls. Thirty seven per cent of respondents were aged between 18 and 34 years old, with 46% aged between 35 and 54 years, and 17% aged over 55. Seventy six per cent of participants were female, and 52% originated from Europe, with 23% coming from Australasia, 13% from North America, 4.1% from South America and 2.1% from Africa. Thirty per cent of participants lived in urban, 30% lived in rural and 40% lived in suburban areas.

#### 2.2.4. Human ethics approval

This study, including the consent procedure, was approved by the University of Queensland Human Ethics Committee (no. 2016001225).

### 2.3. Statistical analyses

Data from the arousal and valence visual analogue scales provided by participants were transformed by SurveyGizmo software into numeric values from 1 to 100.

The demographic variables age, education, as well as respondents' length of experience within each category of experience with chickens, were reclassified as follows: age: 18–34 years, 35–54 years and 55+ years; education: pre-university, undergraduate and postgraduate; and levels of experience with chickens: none, up to 1 year, 2–5 years and over 5 years.

The associations of participants assigning calls to the correct-reward and non-reward context and of the valence and arousal ratings with demographic variables was analysed using generalized mixed models. The association between demographic factors and assignment of calls to the correct context (rewarded or non-rewarded) was analysed using a generalized linear mixed model framework (with correct context assignments denoted by 1 and incorrect by 0), with a logit link function to estimate the odds of rating the calls in the correct context. Linear mixed models were developed to assess the strength of associations between demographic factors and the valence and arousal level ratings provided by participants. Gauss–Hermite quadrature approximation was used for the mixed-effects logistic regression while maximum-likelihood estimation was used for the linear mixed models. Coefficients of the mixed-effects logistic regression were converted into odds ratios and presented with their 95% confidence intervals.

To account for the clustering of measurements within participants and within birds, the participant identity number and the identity of the four different chickens were included as random effect in the models. The call type and the demographic variables were included as fixed effects in all models. We also tested for interactions between demographic variables. Wald tests were used to evaluate the overall significance of variables with more than two levels.

Initially all demographic factors were included together in each of the models. Models were compared using Akaike information criterion scores and likelihood ratio tests to detect which models fitted better than others.

All models were constructed using the Stata SE ([47], Stata Statistical Software: release 18; College Station, TX: StataCorp LLC).

## 3. Results

### 3.1. Effect of demographic variables and experience with chickens on context assignment

**Figure 1.**
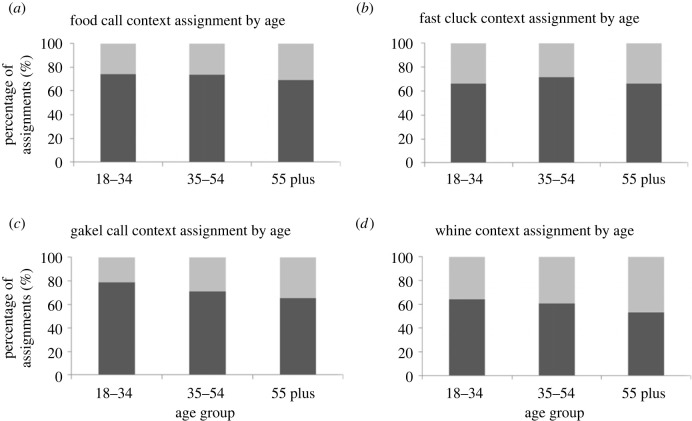
Correct assignment of chicken calls to their context, as either reward-call (food call (*a*), fast cluck (*b*)), or non-reward call (gakel (*c*) and whine call (*d*)). The percentage of participants with correct context assignment by age group is shown in dark grey shading, and the percentage of participants with incorrect assignments by age group is shown in light grey shading.

Overall, participants were able to assign 69% of calls to their correct recording context. Participants performed slightly better at categorizing reward-related calls, with 69% of fast cluck, and 73% of food calls classified correctly, compared with 73% of gakel and 61% of whine calls. For non-reward calls, the percentage of calls that were correctly assigned to the correct context decreased with age (figure 1).

In the final mixed effect logistic regression model (electronic supplementary material, table S1), call type (Wald test *p* < 0.001), age (Wald test *p* = 0.561) and an interaction between these two variables (Wald test *p* < 0.001) were included as fixed effects. Whine calls were significantly less likely to be identified in a correct context (*p* > |z| = 0.009) compared to food calls. For non-reward calls, there was a trend that older participants, compared to younger participants, were less likely to correctly identify that these calls were made in a non-reward context (electronic supplementary material, table S1). Other demographic variables such as experience with chickens, education etc. did not have an effect on assignment of calls to the correct context.

### 3.2. Effect of participant characteristics on valence and arousal ratings

**Figure 2.**
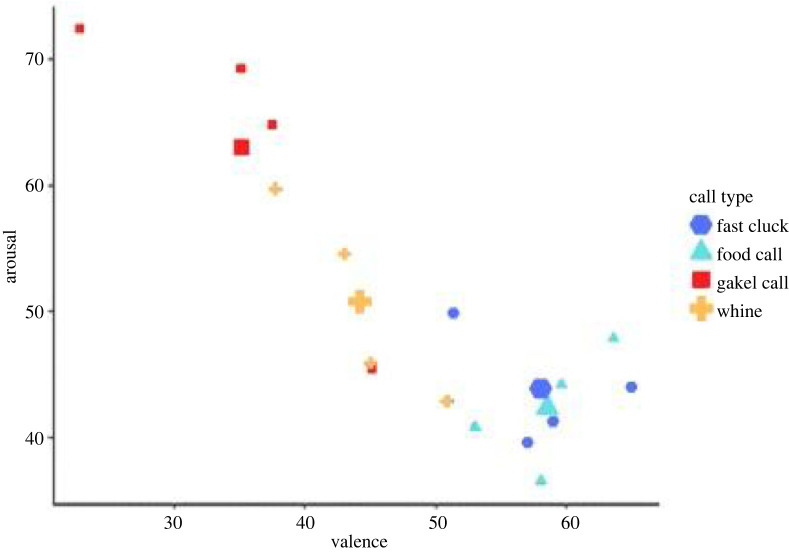
Valence and arousal ratings provided by participants for 16 chicken calls. Larger shapes indicate the mean rating for each call type and smaller shapes indicate individual ratings. Food calls and fast clucks are reward-related calls. Gakel calls and whines are non-reward calls.

The relationship between valence and arousal ratings made by participants is shown in figure 2. Participants rated reward-related calls to be of high positive emotions (high valence) but low intensity or excitement (low arousal). Non-reward gakel calls were rated as representing low valence and high arousal, while the whine call (the other non-reward call) was judged as neutral (around the mid points of about 50 on the 0–100 scale) in terms of arousal and valence (figure 2).

The acoustic structure of calls influenced how participants judged what chickens were experiencing when they produced the calls. Longer calls such as gakel calls (electronic supplementary material, figure S1) represented a more negative emotional state (lower valence) and a higher arousal. Shorter calls (which in general represented reward calls) were rated as representing higher valence and lower arousal levels (electronic supplementary material, figure S1).

**Figure 3.**
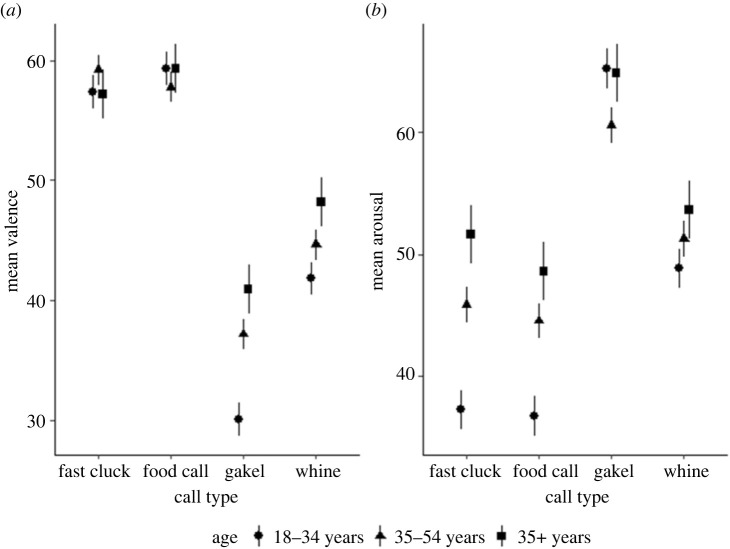
Mean valence (*a*) and arousal level ratings (*b*) with 95% confidence interval for each call type by age group.

Valence rating for reward calls (food calls and fast clucks) were similar across age groups, while the arousal ratings for non-reward calls were also similar across age groups (figure 3).

In the final mixed linear regression models (electronic supplementary material, table S2 and S3) for valence and arousal, call type (Wald test *p* < 0.001, respectively), age (Wald test *p* = 0.661 for valence, Wald test *p* *<* *0.*001 for arousal) and an interaction between these two variables were included as fixed effects (Wald test *p* < 0.001, respectively).

The analysis highlighted that age influenced the evaluations of valence and arousal level of specific call types. Older participants (over 35 years) rated the valence of the non-reward calls (in particular gakel, but also whine calls) as representing greater pleasure than did people in the 18–34-year age group (electronic supplementary material, table S2). By contrast, participants over 35 years old rated the arousal level of the reward-related calls (food calls and fast clucks) higher than did people aged between 18–34 years (electronic supplementary material, table S3).

Other demographic variables such as experience with chickens had no significant effect on valence and arousal ratings (*p* > |z > 0.05).

## 4. Discussion

This study demonstrated that 69% of humans were able to identify whether the chicken vocalizations used in this study were produced in rewarded or non-rewarded contexts, and this ability was not influenced by previous experience with chickens.

Our study reinforces the finding of Filippi *et al*. [36] that the human ability to perceive the emotional content of vocalizations is not restricted to mammals, but extends to other taxa. This suggests that cross-species call recognition may be intrinsic, at least, to vertebrates, and provides evidence that recognition of reward-related calls may be adaptive in some species.

Older people tend to be less adept at identifying the correct context in which calls were made, although this result is more marked in relation to the non-reward-related calls. Whine calls were significantly less likely to be identified in a correct context—whines are wavering, high frequency tonal calls, which may not be perceived as easily as the harsh gakel calls. Greenall *et al*. [26] tested the ability of humans to perceive emotions in the calls of ungulates produced in situations of known emotional arousal and valence and also identified a decrease in correct ratings with age over 20 years.

Furthermore, in our study, older people's ratings of the excitement or arousal levels of reward-related calls were higher than younger people's ratings. In addition, older people rated non-reward calls as representing higher positive emotions or pleasure (higher valence) compared to ratings made by younger people. This difference in age groups may be owing to reduced hearing ability of older people [48–50]. Notably, experience with chickens did not have any effect on calls' context or valence and arousal ratings. This may be because people working with chickens in intensive agriculture do not experience birds that are positively rewarded. Our findings strengthen the evidence that acoustic cues are salient predictors of human recognition of the emotional content of non-human animal vocalizations.

In our study, longer calls were perceived as representing more negative emotions (lower valence) than shorter calls. This finding is consistent with findings on human perception of dog [23,38] and pig [32] vocalizations. By contrast, participants in our study tended to rate longer calls as representing higher excitement (higher arousal) levels.

As we had looped the calls to produce a standard length of 6 s playback time for each call used in the cross-sectional survey, shorter award related calls were played more often in the mp3 files compared to longer non-reward calls. We acknowledge that this may have influenced our results, but we believe that it is important to have the same standard length of sound files played to participants, so that they could listen to each call for the same duration of playback time (and their answers are not biased by having less time when listening to shorter calls).

Another limitation of our study is that we only used eight calls made in a reward and non-reward context (four call types from four chickens, respectively). We acknowledge that valence and arousal are attributes that vary within the same call type of the same chicken, but also between call types of different chickens. However, the focus of the research presented here was not on the identification of call types by participants, but the correct identification of calls made in different contexts. Thus, participants were prevented from knowing the call types used which that may somehow influence their responses. However, we have considered the clustering of sounds made by individual birds and included the bird identity as random effects in the models.

Identifying specific acoustic cues that accurately predict the emotional context of chicken vocalizations was beyond the scope of the research presented here. Future studies should expand on this by testing the effect of other acoustic cues, especially spectral centre of gravity, and harmonic to noise ratio, as well as other frequency and formant-related parameters [15,36]. If future research could identify specific acoustic cues that predict how humans rate arousal in chicken calls, these results could potentially be used in artificially intelligent based detection systems to monitor vocalizations in chickens. Furthermore, if such vocalization monitoring was reliable, it would provide a convenient and cost-effective way to enhance welfare assessment methods in the commercial chicken production industry [51].

This study's predominant finding that a substantial proportion of participants could successfully recognize calls produced in reward-related contexts has important implications for the welfare of farmed chickens. It provides confidence that people involved in chicken husbandry can identify the emotional state of the birds they look after, even if they do not have prior experience. In future research, reward and non-reward related vocalizations could be considered reliable ‘markers' of internal states, allowing for the development of automated assessments of compromised or good welfare states within poultry management systems.

## Data Availability

Data for this project can be accessed here: https://doi.org/10.48610/fa85236 [52]. Supplementary material is available online [53].

## References

[1] Magrath RD, Pitcher BJ, Gardner JL. 2009 Recognition of other species' aerial alarm calls: speaking the same language or learning another? Proc. R. Soc. B **276**, 769. 10.1098/rspb.2008.1368 PMC2660948 19004753

[2] Fant G. 1960 Acoustic theory of speech production. The Hague, The Netherlands: Mouton.

[3] Stein RC. 1973 Sound production in vertebrates: summary and prospectus. Am. Zool. **13**, 1249-1255. (10.1093/icb/13.4.1249)

[4] Titze IR. 1994 Principles of voice production. Englewood Cliffs, NJ: Prentice Hall.

[5] Taylor AM, Reby D. 2010 The contribution of source–filter theory to mammal vocal communication research. J. Zool. **280**, 221-236. (10.1111/j.1469-7998.2009.00661.x)

[6] Gaunt AS, Gaunt SLL, Hector DH. 1976 Mechanics of the syrinx in *Gallus gallus*. A comparison of pressure events in chickens to those in oscines. Condor **78**, 208-223. (10.2307/1366856)

[7] Nowicki S. 1987 Vocal tract resonances in oscine bird sound production: evidence from birdsongs in a helium atmosphere. Nature **325**, 53. (10.1038/325053a0) 3796738

[8] Nesse RM. 1990 Evolutionary explanations of emotions. Hum. Nat. **1**, 261-289. (10.1007/BF02733986) 24222085

[9] Owings DH, Morton ES. 1998 Animal vocal communication: a new approach. Cambridge. UK: Cambridge University Press.

[10] Gogoleva SS, Volodin IA, Volodina EV, Kharlamova AV, Trut LN. 2010 Sign and strength of emotional arousal: vocal correlates of positive and negative attitudes to humans in silver foxes (*Vulpes vulpes*). Behaviour **147**, 1713-1736. (10.1163/000579510X528242)

[11] Rolls ET. 2000 Precis of *the brain and emotion*. Behav. Brain Sci. **23**, 177-234. (10.1017/S0140525X00002429) 11301577

[12] Boissy A et al. 2007 Emotions and cognition: a new approach to animal welfare. Animal Welfare **16**, 37-43. (10.1017/S0962728600031717)

[13] Russell JA. 1980 A circumplex model of affect. J. Pers. Soc. Psychol. **39**, 1161. (10.1037/h0077714)

[14] Mendl M, Burman OHP, Paul ES. 2010 An integrative and functional framework for the study of animal emotion and mood. Proc. R. Soc. B **277**, 2895-2904. (10.1098/rspb.2010.0303) PMC2982018 20685706

[15] Briefer EF. 2012 Vocal expression of emotions in mammals: mechanisms of production and evidence. J. Zool. **288**, 1-20. (10.1111/j.1469-7998.2012.00920.x)

[16] Brudzynski SM. 2007 Ultrasonic calls of rats as indicator variables of negative or positive states: acetylcholine–dopamine interaction and acoustic coding. Behav. Brain Res. **182**, 261-273. (10.1016/j.bbr.2007.03.004) 17467067

[17] Taylor AM, Reby D, McComb K. 2009 Context-related variation in the vocal growling behaviour of the domestic dog (*Canis familiaris*). Ethology **115**, 905-915. (10.1111/j.1439-0310.2009.01681.x)

[18] McGrath N, Dunlop R, Dwyer C, Burman O, Phillips CJ. 2017 Hens vary their vocal repertoire and structure when anticipating different types of reward. Anim. Behav. **130**, 79-96. (10.1016/j.anbehav.2017.05.025)

[19] Pongrácz P, Molnar C, Miklosi A, Csanyi V. 2005 Human listeners are able to classify dog (*Canis familiaris*) barks recorded in different situations. J. Comp. Psychol. **119**, 136-144. (10.1037/0735-7036.119.2.136) 15982157

[20] Pongrácz P, Molnár C, Miklósi Á. 2006 Acoustic parameters of dog barks carry emotional information for humans. Appl. Anim. Behav. Sci. **100**, 228-240. (10.1016/j.applanim.2005.12.004)

[21] Molnár C, Pongrácz P, Miklósi á. 2010 Seeing with ears: sightless humans’ perception of dog bark provides a test for structural rules in vocal communication. Q. J. Exp. Psychol. (Colchester) **63**, 1004-1013. (10.1080/17470210903168243) 19760535

[22] Pongrácz P, Molnár C, Dóka A, Miklósi Á. 2011 Do children understand man's best friend? Classification of dog barks by pre-adolescents and adults. Appl. Anim. Behav. Sci. **135**, 95-102. (10.1016/j.applanim.2011.09.005)

[23] Faragó T, Andics A, Devecseri V, Kis A, Gácsi M, Miklósi Á. 2014 Humans rely on the same rules to assess emotional valence and intensity in conspecific and dog vocalizations. Biol. Lett. **10**, 20130926. (10.1098/rsbl.2013.0926) 24402716 PMC3917336

[24] Tallet C, Spinka M, Maruscakova I, Simecek P. 2010 Human perception of vocalizations of domestic piglets and modulation by experience with domestic pigs (*Sus scrofa*). J. Comp. Psychol. **124**, 81-91. (10.1037/a0017354) 20175599

[25] Filippi P, Gogoleva SS, Volodina EV, Volodin IA, Boer B. 2017 Humans identify negative (but not positive) arousal in silver fox vocalizations: implications for the adaptive value of interspecific eavesdropping. Cur. Zool. **63**, 445-456. (10.1093/cz/zox035) PMC5804197 29492004

[26] Greenall JS, Cornu L, Maigrot A-L, de la Torre MP, Briefer EF. 2022 Age, empathy, familiarity, domestication and call features enhance human perception of animal emotion expressions. R. Soc. Open Sci. **9**, 221138. (10.1098/rsos.221138) 36483756 PMC9727503

[27] Magrath RD, Haff TM, Fallow PM, Radford AN. 2015 Eavesdropping on heterospecific alarm calls: from mechanisms to consequences. Biol. Rev. **90**, 560-586. (10.1111/brv.12122) 24917385

[28] Yin S, McCowan B. 2004 Barking in domestic dogs: context specificity and individual identification. Anim. Behav. **68**, 343-355. (10.1016/j.anbehav.2003.07.016)

[29] Siebert ER, Parr LA. 2003 A structural and contextual analysis of chimpanzee screams. Ann. N Y Acad. Sci. **1000**, 104. (10.1196/annals.1280.022) 14766626

[30] Compton L, Clarke J, Seidensticker J, Ingrisano D. 2001 Acoustic characteristics of white-nosed coati vocalizations: a test of motivation-structural rules. J. Mammal. **82**, 1054-1058. (10.1644/1545-1542(2001)082<1054:ACOWNC>2.0.CO;2)

[31] Feighny J, Williamson K, Clarke J. 2006 North American elk bugle vocalizations: male and female bugle call structure and context. J. Mammal. **87**, 1072-1077. (10.1644/06-MAMM-A-079R2.1)

[32] Maruščáková IL, Linhart P, Ratcliffe VF, Tallet C, Reby D, Špinka M. 2015 Humans (*Homo sapiens*) judge the emotional content of piglet (*Sus scrofa domestica*) calls based on simple acoustic parameters, not personality, empathy, nor attitude toward animals. J. Comp. Psychol. **129**, 121. (10.1037/a0038870) 25798794

[33] Andics A, Gácsi M, Faragó T, Kis A, Miklósi Á. 2014 Voice-sensitive regions in the dog and human brain are revealed by comparative fMRI. Curr. Biol. **24**, 574-578. (10.1016/j.cub.2014.01.058) 24560578

[34] Lingle S, Riede T. 2014 Deer mothers are sensitive to infant distress vocalizations of diverse mammalian species. Am. Nat. **184**, 510-522. (10.1086/677677) 25226186

[35] Albuquerque N, Guo K, Wilkinson A, Savalli C, Otta E, Mills D. 2016 Dogs recognize dog and human emotions. Biol. Lett. **12**, 20150883. (10.1098/rsbl.2015.0883) 26763220 PMC4785927

[36] Filippi P et al. 2017 Humans recognize emotional arousal in vocalizations across all classes of terrestrial vertebrates: evidence for acoustic universals. Proc. R. Soc. B **284**, 20170990. (10.1098/rspb.2017.0990) PMC5543225 28747478

[37] Boissy A et al. 2007 Assessment of positive emotions in animals to improve their welfare. Physiol. Behav. **92**, 375-397. (10.1016/j.physbeh.2007.02.003) 17428510

[38] Faragó T, Takács N, Miklósi Á, Pongrácz P. 2017 Dog growls express various contextual and affective content for human listeners. R. Soc. Open Sci. **4**, 170134. (10.1098/rsos.170134) 28573021 PMC5451822

[39] Nicastro N, Owren MJ. 2003 Classification of domestic cat (*Felis catus*) vocalizations by naive and experienced human listeners. J. Comp. Psychol. **117**, 44-52. (10.1037/0735-7036.117.1.44) 12735363

[40] Linnankoski I, Laakso M, Aulanko R, Leinonen L. 1994 Recognition of emotions in macaque vocalizations by children and adults. Lang. Commun. **14**, 183. (10.1016/0271-5309(94)90012-4)

[41] Scheumann M, Hasting A, Kotz S, Zimmermann E. 2014 The voice of emotion across species: how do human listeners recognize animals' affective states? PloS ONE **9**, e91192. (10.1371/journal.pone.0091192) 24621604 PMC3951321

[42] Collias N, Joos M. 1953 The spectrographic analysis of sound signals of the domestic fowl. Behaviour **5**, 175-188. (10.1163/156853953X00104)

[43] Evans CS, Evans L. 1999 Chicken food calls are functionally referential. Anim. Behav. **58**, 307-319. (10.1006/anbe.1999.1143) 10458882

[44] Marx G, Leppelt J, Ellendorff F. 2001 Vocalisation in chicks (*Gallus gallus dom*.) during stepwise social isolation. Appl. Anim. Behav. Sci. **75**, 61-74. (10.1016/S0168-1591(01)00180-0)

[45] Adobe Systems Inc. 2013 Adobe audition CC. San Jose, CA. See https://www.adobe.com/au/products/audition.html.

[46] Alchemer. 2017 SurveyGizmo. Louisville, CO. See https://www.alchemer.com/.

[47] StataCorp. 2023 Stata statistical software: release 18. College Station, TX: StataCorp LLC.

[48] Huang Q, Tang J. 2010 Age-related hearing loss or presbycusis. Eur. Arch. Otorhinolaryngol. **267**, 1179-1191. (10.1007/s00405-010-1270-7) 20464410

[49] Ruffman T, Morris-Trainor Z. 2011 Do dogs understand human emotional expressions? J. Vet. Behav. **6**, 97-98. (10.1016/j.jveb.2010.08.009)

[50] Amorim M, Anikin A, Mendes AJ, Lima CF, Kotz SA, Pinheiro AP. 2021 Changes in vocal emotion recognition across the life span. Emotion **21**, 315-325. (10.1037/emo0000692) 31647283

[51] Mao A et al. 2022 Automated identification of chicken distress vocalizations using deep learning models. J. R. Soc. Interface **19**, 20210921. (10.1098/rsif.2021.0921) 35765806 PMC9240672

[52] Henning J. 2023 Data from: Humans can identify reward-related call types of chickens. The University of Queensland Research Data Manager. (10.48610/fa85236)

[53] McGrath N, Phillips CJC, Burman OHP, Dwyer CM, Henning J. 2024 Humans can identify reward-related call types of chickens. Figshare. (10.6084/m9.figshare.c.6980764) PMC10762433 38179075

